# “Even Though We Have Different Colors, We Are All Equal Here”: Immigrants building a sense of community and wellbeing through sport participation

**DOI:** 10.1002/jcop.22897

**Published:** 2022-06-18

**Authors:** Chiara Corvino, Sara Martinez‐Damia, Mattia Belluzzi, Daniela Marzana, Chiara D'Angelo

**Affiliations:** ^1^ Department of Psychology Università Cattolica del Sacro Cuore Milan Italy

**Keywords:** community participation, immigrants, psychological sense of community, social change, sport, sport‐for‐development, subjective well‐being

## Abstract

Although there are several studies reporting the beneficial role of sports in immigrant health, more research is needed to understand whether and how these activities can guide the psychological sense of community (SOC) and well‐being outcomes. This study aims at exploring how sport participation among immigrants contributes to developing their SOC and subjective well‐being. We analyzed the experiences of 17 immigrants participating in Balon Mundial (BM), an annual multicultural football tournament in Turin (Italy). A thematic deductive theory‐driven analysis was implemented based on in‐depth interviews. BM developed a psychological SOC by providing immigrants with a safe space—based on norms of accessibility and fair play—to mutually share traditions while forming positive and trusting bonds. All these dimensions of the tournament were connected to an enhancement of immigrants' subjective well‐being in terms of happiness, self‐care, sense of acceptance and cultural intelligence. Sport participation can sustain immigrants' psychological SOC and subjective well‐being under specific conditions: (a) when norms are developed based on inclusion; and (b) when a shared goal and  history are built among participants.

## 1 INTRODUCTION

Community participation is one way for marginalized groups to find a sense of belonging and to advocate for their needs (Martin Barò, 1986; Montero & Sonn, 2009). The literature on social and community psychology has underlined a link between community participation, psychological sense of community (SOC) and subjective well‐being (Mannarini & Fedi, 2009; Musick & Wilson, 2003; Soukiazis & Ramos, 2016; Talò et al., 2014). Research with immigrants has found that participation within associations or organized groups has positive effects on subjective well‐being (Alfieri et al., 2019; Marzana, Alfieri, et al., 2020; Marzana, Martinez‐Damia et al., 2020; Taurini et al., 2017) via a psychological SOC (Ramos et al., 2017, 2020). Indeed, immigrant participation fosters belonginess (Guo, 2014), social capital (Handy & Greenspan, 2009) and feelings of acceptance (Sonn & Fisher, 1996), thus enhancing immigrant subjective well‐being. Following other scholars who studied dance as a form of community participation for immigrants (see, e.g., Marzana et al., 2019; Marzana, Martinez‐Damia et al., 2020), the current study conceptualizes sport as a specific form of community participation. Results on immigrant community participation have been found to be similar to those on immigrants who play sports (Fader, 2018; Stone, 2018; Whitley et al., 2016), although with some open concerns. On the one hand, a number of studies underline the positive influence of sport on immigrants' psychological SOC and well‐being (Alfieri et al., 2021; Fader, 2018; Nathan et al., 2013; Stone, 2018; Whitley et al., 2016). On the other hand, other studies report that sport activities entail risks of discrimination and social marginalization of immigrants (Bradbury, 2013; Spaaij, 2012). As a consequence, the role of sport remains controversial and more investigation is needed, especially regarding the specific conditions under which it may effectively promote immigrants' psychological SOC and well‐being (Alfieri et al., 2021; D'Angelo et al., 2021, 2017; Whitley et al., 2019).

The present work aims to describe the psychological SOC that immigrants experience through sport participation and how their SOC is linked to their subjective well‐being. After presenting a review of the literature regarding these three variables, we will describe a qualitative study that was implemented in partnership with an Italian non‐governmental organization (NGO) that organizes an annual soccer event for immigrants. We conclude with the results and discussion and also provide some practical implications.

### 1.1 Community participation, subjective well‐being, and psychological SOC

The engagement of immigrants in community life can be referred to as community participation (Gele & Harsløf, 2012). Community participation can take several forms: Volunteering, sport participation, civic and social engagement, cultural or proenvironmental behavior, political participation, and religious service. Community participation usually takes shape within nonprofit entities, such as formal and informal associations, that generally aim to develop socialization and a sense of belonging for their members. According to the psychology of liberation, community participation is a strong tool for social change because it provides people who are usually socially excluded the opportunity to access resources in their organization within a group context (Martin Barò, 1986; Montero & Sonn, 2009). Qualitative studies have shown that community participation among immigrants improves their bicultural competences, social relationships, ethnic identity and adaptation to the new culture (Handy & Greenspan, 2009; Marzana, Martinez‐Damia et al., 2020; Taurini et al., 2017). Alfieri et al. (2019) reported that immigrants who participate in the community have higher levels of subjective well‐being than those who do not participate. In the present study, we will refer to Prilleltensky's (2012) conceptualization of well‐being as a multidimensional construct characterized by the simultaneous and balanced satisfaction of needs across six domains: economic, physical, occupational, psychological, community, and interpersonal.

Some authors have focused on the role of psychological SOC as a mediator of the relationship between community participation and subjective well‐being (Hombrados‐Mendieta et al., 2013; Ramos et al., 2017, 2020; Sonn & Fisher, 1996, 1998). Psychological SOC has been defined as “the feeling that members have a sense of belonging, that they are important to each other and to the group, that they have a shared belief about whether their needs will be met through the commitment of being together” (McMillan & Chavis, 1986; p. 9). García‐Cid et al. (2020), studying immigrants from Eastern Europe, Africa and Latin America who live in Malaga, found that a psychological SOC may reduce the impact of perceived discrimination on subjective well‐being (García‐Cid et al., 2020). Therefore, psychological SOC seems to be a central variable for planning interventions that aim to increase the subjective well‐being of the immigrant population. According to McMillan (1996), psychological SOC comprises four dimensions: (a) Spirit, which refers to a “spark” (p. 318) that begins with one's sense of belonging to a group and is rooted within the faith that one will be welcome and accepted; (b) Trust, which refers to a “flame” (p. 318), which can become a fire if there is an organizational structure (norms, rules and decision‐making conduct) that allows people to know what to expect and to develop a sense of mastery; (c) Trade, which refers to self‐disclosure (i.e., the sharing of feeling between members) and exchanging of resources; and (d) Art, which refers to a shared history that represents traditions, symbols, and values that reinforce the spirit of that community.

### 1.2 Sport and immigrants: Benefits and settings

Several studies have reported that sport can promote a wide range of nonsporting outcomes for people living in conditions of vulnerability, including subjective well‐being and psychological SOC (D'Angelo et al., 2019, 2020, 2021; Kumar et al., 2018; Nathan et al., 2010; Woodhouse & Conricode, 2017). In the case of immigrants, sport can strengthen ethnic identity, reduce stress and negative thoughts, and increase self‐efficacy (Dukic et al., 2017; Marzana, Martinez Damia et al., 2020). It also helps immigrant to build a life routine, to regain control when facing the migration‐related uncertainties (Hartley et al., 2017) and to create positive relationships (Fader, 2018; Nathan et al., 2013; Stone, 2018; Whitley et al., 2016). For example, Alfieri et al. (2021) underlined that those immigrants who participate in sports have higher levels of both subjective well‐being and psychological SOC compared to immigrants who participate in other forms of community activities (e.g., volunteering).

Although sport usually takes place within supportive settings that sustain immigrants' inclusion (Olliff, 2008), many authors (Anderson et al., 2019; Block & Gibbs, 2017; Hatzigeorgiadis et al., 2013; McDonald et al., 2019; Nowy et al., 2020; Sanchèz et al., 2013) have stressed the importance of studying the organizational, policy‐driven and structural aspects of sport programs. Although it is recognized that sport is way for immigrants to fully enter the social life of the new country in which they live, we also acknowledge that sport is “not a panacea to promote belonging and cohesion” (Spracklen et al., 2015; p. 2) because it may also lead to experiences of discrimination and violence (Mauro, 2016). In the words of Sonn (2002), some settings within communities “may also be construed as exclusionary [and] only by understanding the nature of the settings and the functions they fulfill, will we be able to optimize their potential for people and reduce the potential for harm following exclusion” (p. 218). For this reason, it is important to study specific experiences (McDonald et al., 2019; Nathan et al., 2010) and to listen to the direct experiences of the immigrants who participate directly in those settings (Müller et al., 2008; Warner et al., 2012).

In this study, we combine the literature of community psychology with that of sport, and we use the term “sport participation” to refer to the particular type of community participation that uses sport as a tool to be part of the community. Research suggests that sport can have similar effects to other forms of participation, but more evidence is needed to establish this link and to understand how sport can increase immigrants' psychological SOC and subjective well‐being. Specifically, we aim to explore the physical, psychological, community and interpersonal domains of immigrant subjective well‐being (Prilleltensky, 2012).

### 1.3 The present study

The present study was conducted in Italy, where immigrants have always encountered a policy marginalizing them through laws based on flow regulation and emergency (Colucci, 2018) and through media propaganda that has framed them as security issue over a period of almost two decades (Mazzara et al., 2020). According to Italian scholars (Ambrosini, 2013; Caneva, 2014) Italian legislation has implemented continuous and structural exclusion of immigrants. Especially in Northern Italy. Moreover, levels of intolerance toward immigrants have increased in recent years (Eurispes, 2020).

A collaboration was developed with an Italian NGO, namely Balon Mundial (BM) ASD Onlus, which is based in Turin (Northern Italy). It implements football‐based interventions for immigrants and organizes an annual summer event called “Balon Mundial” (BM). This event consists of two soccer tournaments (one for males and one for females) with teams of immigrants. Its main purpose is to provide a space where immigrants and Italians can meet and form new bonds, with the ultimate goal of overcoming prejudices, sharing and building a sense of belonging. The event involves people from fifty different countries (from the regions of Eastern Europe, South America, Africa, and the Middle East) living in Turin. Teams are usually ethnically homogeneous (i.e., they gather immigrants coming from the same countries in the same team). Each team is run by an immigrant leader and trains itself during the year. BM is structured with the same format as the FIFA World Cup, although it has specific rules that differ from the ones provided by FIFA. Specifically, the criteria for winning the match do not include only the number of goals per team but also an evaluation of the team behavior during the competition. Unfair and violent behaviors are strongly sanctioned and may compromise the winner even if the team has accumulated more goals than its rivals. Moreover, during the soccer event, culinary booths sell traditional foods to the public and offer music and cultural traditions of each country.

The aims of the present work were to: (a) explore the creation of the psychological SOC within the soccer event and (b) explore what dimensions of psychological SOC were connected to the subjective well‐being of immigrants who participate.

## 2 METHOD

### 2.1 Participants

The tournament involves approximately eighteen teams each year for the male tournament, six teams for the female one, and around 1000 participants including team players and supporters. People who have participated in the tournament for the last 3 years came from Italy, Brazil, Colombia, Peru, Bolivia, Ivory Coast, Gambia, Iraq, Mali, Nigeria, Russia, Lebanon, and Romania for the male teams and Italy, Brazil, Iran, Colombia, Peru, and Senegal for the female teams. The creation of multiple teams representing the same country is allowed, especially when many people of the same nationality want to participate in the event.

In agreement with the NGO, we decided to invite to the research the most permanent teams, as they are considered the pillars of the tournament. Furthermore, for the purposes of this study, we decided to focus our attention on the experiences of the immigrant leaders of the team (*N* = 12) and the volunteers managing food banquets (*N* = 2). This choice was guided by the following reasons. First, the team leaders have a key role within the tournament, as they are the representative of the team for bureaucracy issues and subscriptions as well the ones allowed to speak to the referee when disputes occur during matches. Moreover, leaders are generally strongly engaged in the event and are recognized by other players. Hence, we selected them as key informants. We decided to exclude the Italian leaders because we were interested in exploring SOC and well‐being specifically for immigrants. On the other hand, the NGO board recommended that we involve volunteers for food as essential figures to gain a more comprehensive view of the tournament. In total, 17 tournament participants (8 women, 9 men) between the ages of 27 and 60 were interviewed. More demographic information is outlined in Table 1.

**Table 1:** Demographic information of participants

Code	Role	Sex	Age	Country of origin	Educational level	Years in Italy	Legal document	Job
01	L	Female	27	Brasil	Master	3	In process of regularization	Caregiver for elderly
02	L	Female	31	Brasil	Master	14	Italian citizenship	Sports trainer
03	L	Female	46	Colombia	Bachelor	22	Long‐term residence card	Caregiver for elderly
04	L	Female	50	Ecuador	Middle school	19	Long‐term residence card	Babysitter
05	L	Female	48	Ecuador	Master	19	Long‐term residence card	Nurse
06	L	Male	45	Bolivia	NS	16	Long‐term residence card	Courier
07	L	Male	34	Colombia	Master	16	Long‐term residence card	Workman
08	L	Male	38	Ivory coast	NS	22	Long‐term residence card	Employed but the field not specified
09	L	Male	60	Ecuador	Master	24	Work residence permit	Workman
10	L	Male	26	Guinea	Professional bachelor	5	Not specified	Employed but the field not specified
11	L	Male	36	Iraq	PhD	11	Work residence permit	Engineer
12	L	Male	64	Peru	NS	NS	Long‐term residence card	Disability pension
13	F	Female	43	Brasil	Bachelor	22	Long‐term residence card	Babysitter
14	F	Male	33	Moldova	Professional bachelor	17	Long‐term residence card	Company owner
15	M	Female	31	Iran	Master	4	Visa student	Student
16	M	Female	29	Iran	Master	6	Visa student	Engineer
17	M	Female	30	Iran	Master	NS	Visa student	Student

Abbreviations: F, food volunteer; L, leader of the team; M, member of the team, NS, not specified.

### 2.2 Interview

We interviewed immigrants using a semi‐structured guide that explores: (a) migration history and current living situations; (b) the tournament and motivation to participate; (c) dimensions of SOC in relation to the tournament; (d) feeling of discrimination both within and outside the tournament; and (e) subjective well‐being in the tournament. All participants were contacted by two field researchers and provided informed consent before the interviews. Because of the COVID‐19 pandemic restrictions, the interviews were done online. Different digital apps and platforms (Whatsapp, Zoom, and Teams) were used to meet participants' needs, safeguarding in all cases the possibility for the researcher and participants to see each other and to make an audio recording of the interview. Each interview lasted between 45 and 75 min. Two researchers with previous experience in qualitative methods conducted the interviews in Italian, Spanish or English according to the preference of participants. Interviews were transcribed verbatim in Italian by a third researcher. To protect the privacy of interviewees, we assigned an identification number to each participant.

### 2.3 Procedures and data analysis

The research team had already collaborated with the NGO in previous projects, building mutual trust and confidence. A presentation of the research was forwarded through the NGO channels to all its immigrant members who were invited to participate (purposive sampling). As a complementary selection strategy, we also asked each interviewee to indicate another potential participant (snowball technique). Participation was totally free, and no reward was provided. Because of the outbreak of the COVID‐19 pandemic, the event did not take place in 2020, and the NGO had the opportunity to listen to the experiences of their immigrant members. The research took place between January and April 2021 and used a qualitative methodology that would allow participants to share flexibly their memories and experiences of the event. The study protocol was approved by the Ethical Institutional Board of the University (no.  protocol 16−21).

The analysis of the transcripts of interviews was based on a theoretical/deductive thematic analysis (Braun & Clarke, 2006). Deductive coding was used to explore SOC and well‐being according to the theoretical models described in the introduction. First, we developed a coding manual based on the four dimensions of SOC (Spirit, Trust, Trade, and Art) and the four domains of subjective well‐being (physical, psychological, community, and interpersonal). Then, three researchers engaged in reading all the transcripts to test the reliability of the coding manual. A revised version of the manual was created, and the researcher who had previously engaged in transcribing the interviews analyzed all of them according to the manual. During frequent debriefing sessions, the two field researchers acted as supervisors, providing suggestions, and insights. These sessions were used to ensure trustworthiness and to share a communal meaning as recommended by Shenton (2004). When a final set of codes was developed, the three researchers defined together how to cluster codes into themes (psychological SOC and subjective well‐being). During this process, many memos were taken to detect initial relationships between the two main constructs of the research. We considered memos a fundamental part of the coding process because they provided us with a storyline to justify our choices during data analysis (Stuckey, 2015). Specifically, we jotted down the co‐occurrences of certain codes using our reflexivity as an active part of data construction that helped us to better capture the meanings given by the participants to BM (Braun & Clarke, 2019). We realized that participants often reported the positive impact of BM in terms of well‐being by connecting it to different aspects of SOC. Indeed, certain codes (e.g., “sense of belonging”; “sport team”; “positive bonds”) could be transversally included in diverse themes related to SOC and well‐being. Finally, we corroborated themes and connections by reviewing all the previous stages of our analysis, by labeling and choosing the most significant quotations to present the findings. Scrutiny with peers who were experts in the field of community participation of immigrant people and in sport projects was used as another method to ensure credibility (Shenton, 2004).

## 3 RESULTS

The results show that during the event, immigrants perceive a psychological SOC that is connected to their well‐being.

### 3.1 Psychological SOC

Table 2 describes the themes that we created for each dimension of the psychological SOC.

**Table 2:** Themes and quotations for the four dimensions of psychological sense of community

PSOC	Themes	Quotations
Spirit	Commitment	In the proximity of the tournament, we usually do a mini preparation of one month, where we do some training (…) when the organizer arranges a meeting and tells me “Ok, let's do BM this year”, then I take in some people and we start to prepare it, two, three months before. (Male leader)
	Belonging	It's a team that I like very much, I put my heart into this team because they are good people that I am happy to know. (Female leader)
	A stage for one's own ethnic origins	I am proud to represent my country and to know that even if we are far away, we are always with our flag up, always with our tricolor; we participate with our flag and represent our country, knowing that our country is a country of culture, a rich country… I think any person from a country wants to love his/her country and want to show how great it is, whatever place it is. (Female leader)
Trade	Multiculturalism	Sport is the purpose for which you compete with the team, but then at the end there are many typical things behind it, many things that allow you to know many cultures, both for us, for the Italians, and for the guys of other nationalities. So, you learn a little bit about all the foreign communities that are in Turin. (Male leader)
	Building social network	You realize that there are people who care for us, there is no selfishness, also because they are Italians who take care of us foreigners. (Female leader)
Art	The ritual of food	Each nationality also has its own stand, those who want to join, in which they also bring food… the more homemade ones, understand? The typical foods of each nationality and each country, so you find the stand of Colombia, Brazil, Congo, Nigeria, and each nationality sells its typical foods. Then you can understand… the other nations by the way they eat, the way they cook. (Male leader)
	Making oneself visible	And the only motivation to participate in this is to show who we are, Ecuador, as a culture, as a setting (…) because I am proud of my country and I am proud of my family. It's a way to represent the Ecuadorian family. (Male leader)
Trust	Accessibility and fair play	It is not a professional tournament, and that makes so much difference because there you play for the pleasure of playing. You don't play for the money or for the trophy, but you play because you have pleasure in playing, that's really the true meaning of the word play.
		The tournament is based in fairplay.

#### 3.1.1 Spirit

Within the soccer event, spirit is characterized by three themes: commitment, belonging and a stage to show one's own ethnic origins. Commitment refers to participants' strong investment in the tournament. The event is perceived as part of their summer routine, and they look forward to it: “Every year it is something nice (…) it is a kind of hope, something nice coming in the summer where we can all meet” (Male leader). Interviewees show a lot of effort in getting physically prepared months before the event, spending a lot of time in exercise. As a result, those who take part in BM bring their maximum potential to the event: “Since we know it [the event] starts in June, we start calling each other in March, we create a [WhatsApp] group [where we say] ‘guys, let's start training a little bit’ because we basically start training 3 months before” (Female leader). Members of the teams are willing to make themselves available to one another and are emotionally engaged in the event.

Belonging refers to participants' sense of attachment to the event. Specifically, participants described how BM contributes to building a sense of belonging which extends from the family of origin to own's own team and then to other teams as well. Interviewees indeed reported that tournament is an occasion to bring the family together with some people playing and others cheering: “It is a kind of a family party, because we go with the family. I do not go by myself but with my children, with my wife, with the dog… so it should be a family party” (Male leader). The possibility to mix the support for relatives and for one's country of origin strengthens participants' attachment to the event. Leaders and players most identify themselves as members of the teams when talking about the tournament, and many reported the bond of closeness with their team members, a relationship that often turns into friendship and leads to informal meetings outside the event:

*We have this group [a WhatsApp group comprising all the team members] where we organize birthdays, organize outings, meals at someone's house. When there is a collection for someone's birthday, we organize, we are more like friends, outside the field we are friends, a family*. (Male leader)

Lastly, all interviews report how BM contributes to defining new social ties beyond one's own team. These bonds are described as positive and stable over time and as sources of emotional security for those who take part in the event. “It is a way to know and interact with other nationalities, because from the tournament new friendships have been formed and last across the years (…) you develop friendship and knowledge that in other contexts you would never have” (Male leader).

The last category for the dimension of Spirit, which is strictly linked to commitment and belonging, refers to BM as a stage for one's own ethnic origins. BM is described by participants as a place where people of different countries can give visibility to their cultural and national specificities. The event is perceived as a stage where immigrants can show their ethnic identities and pride. As one team leader explains, participating in the event allows immigrants to witness their national belonging and strengthen their ties with their community of origin:

*You represent your nation and in that moment there, your nationality comes out, your roots: I am Brazilian, Peruvian and so on, so I represent my country in this moment and so I give the best of me and this is the difference compared to other tournaments in my opinion and compared to professional sport*. (Female leader)

#### 3.1.2 Trust

The event is based on two main norms: (a) accessibility and (b) fair play. Accessibility refers to the possibility to participate to the tournament regardless of economic resources, legal status and sport ability. Indeed, BM is affordable because, unlike other tournaments, it is not necessary to pay an entry fee to participate. Moreover, BM is legally accessible because, unlike federal competitions where documents and residence permits are generally required, is opened to anyone regardless of their legal status. Finally, BM is accessible to every type of player, from those with great experience in football to those approaching the discipline for the first time:

*You don't have to pay or show documents. It is nice because you can see different levels, both for boys and girls. Some teams are very organized, so you can see technically well played football. Other teams are just there for the pleasure of being together, so they come*. (Male leader)

The second norm is fair play. During the matches, points are deducted or added to the teams according to the number of fouls committed or bad behaviors shown (e.g., insults/violent reactions during the match). This particular rule makes the event different from other similar football tournaments. Because of this norm, participants and supporters are more likely to maintain a respectful and positive climate during the competition.

*For example, in 2016 we came second and in the stands of the stadium there were people jumping, shouting. The fans of Cameroon, we played the final against Cameroon, were shouting… it was a kind of match between supporters who shouted the loudest, there was half Cameroon and half our supporters, there was the music, people who brought musical instruments… another atmosphere, another thing… it is not the same as a normal league game*. (Male leader)

The focus on fair play may be the reason why participants largely reported they have never encountered discrimination either within or outside the tournament: “all the people here are nice. I don't see discrimination. All people respect other's countries (Male leader).

#### 3.1.3 Trade

Trade refers to self‐disclosure and resource exchange within the community. During this event, trade occurs because of the promotion of multiculturalism and the building of a social network.

The exchange between multiple cultures is described as a distinctive element of the tournament that allows participants to move beyond stereotypes and create a sense of reciprocity and trust among people taking part in the event. Being side by side with people from different cultures and nationalities, during the competition and the moments surrounding it (e.g., the food banquets), makes participants more familiar with what is initially unknown to them:

*You begin to have a different perspective (….) An African or a Colombian is seen by 60% of people as a drug trafficker, the one who sends the drugs to Europe. Instead, [within BM], you get to know all the other faces of each nationality, you see them differently (…) you can understand that [in] reality it is not like that*. (Male leader)

*Being close to other nationalities makes participants develop a sense of equality and mutuality where participants can recognize that they are all human: there we are the same even though we have different colors* (Female leader).

Furthermore, the tournament promotes the formation of caring and significant bonds that become a source of social support. Becoming part of the team allows those who have just arrived in Italy to create new ties that can help them with their integration in the new country. Moreover, the team allows immigrants who are already integrated to take care of those who are still at the beginning of their migration path using the experience they have acquired:

*There are a lot of them now in the group. Six years ago, they didn't know anyone [he is talking about the members of his team], they didn't have any parents or anyone here. So, I took them in, I introduced them to some friends, now they are members of that group, they have papers, they are working. (…) When I see a person that's just come in, before he goes and does something stupid, we try to integrate him into the group and give him some right advice, some right tips. Maybe he doesn't get his papers, he starts freaking out, until he finds the right way to find what he was looking for, we are behind him, we give him a hand. It's not just soccer*. (Male leader)

This dimension of exchange and mutual support also emerged in other interviews, especially regarding job search. Several participants reported they were able to find employment through the event's network: “It [the event] also opened doors for me for work (…) by knowing people [within the tournament], I found work” (Female leader).

#### 3.1.4 Art

The tournament is described by all the interviewees as a great party in which soccer becomes a way of staying together and getting closer to new cultures. Alongside the sporting event, the food banquets are mentioned as a crucial element that guides the creation of a climate of conviviality. Tasting the food of other nationalities is perceived as a ritual of exchange and mutual understanding between communities. This tool has a dual value. On the one hand, food becomes a means to open up to others. On the other, rediscovering the flavors of one's own nation allows one to preserve one's bond with the motherland, as explained by this interviewee:

*From sweets to green mango cakes, you manage to find these fruits that you [have lost] the habit of eating (…) because it's now a different world where you need to adapt…. Being here [in the tournament] reminds you a bit of home, it's like being gossiping ladies, you go to peek at other people's houses, what they do, what they don't do, what they cook, what they don't cook. Even out of curiosity… you pass by the Nigeria stand, for example, you see something tasty and you say “I'll try it”. The same thing applies to them and everyone else*. (Male leader)

Furthermore, the event itself is perceived as a ritual that provides immigrants with the opportunity to show themselves as positive people to Italians and other participants: “I believe that the first thing to break down [the prejudice] is not to isolate ourselves. Show ourselves (…) And the only motivation to participate in this is to show who we are: we are Ecuador” (Male leader). The event is described as a collective experience in which participants can challenge stereotypes through the means of sport. Taking the role of a football player in a national team enables one to show one's identity and culture by using the common positive language of football:

*This tournament helps us a lot, because it gives us the opportunity to be seen (…) So, we're not just Brazilians doing who knows what, or Moroccans doing who knows what, but we're the players of that nation. This breaks down the preconceptions from that point of view*. (Female leader)

### 3.2 Linking psychological SOC to domains of subjective well‐being

**Figure 1:**
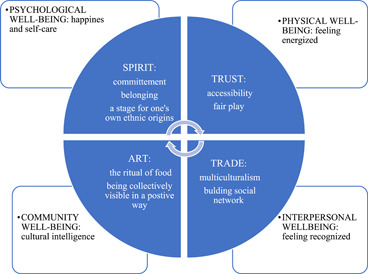
Overview of the results

Figure 1 shows how the dimensions of psychological SOC are connected with some domains of subjective well‐being.

We find that the event promotes the subjective well‐being among immigrants in four out of six domains: psychological, physical, interpersonal, and community. Regarding psychological well‐being, the spirit dimensions developed during the event bring immigrants happiness. BM represents a way to fight loneliness because it fills the weekends: “it's a way to spend your Sundays” and to have a weekend “out of the ordinary.” For some, even the expectation of the tournament itself brings happiness:

*When Saturday or Sunday comes, we're all excited that we have to go play (…) Since BM started, I'm always happier, I'm waiting to be able to play. In that sense it has changed me, I always wish it doesn't end, you get up in the morning happy that you have to go play*. (Male leader)

Many people describe BM as time off, where they can have fun and get away from the problems they have in their lives: “when I'm on the pitch I forget everything bad that happens outside” (Female leader). Sport represents a way for immigrants to take care of themselves and provides a space, especially for immigrant women, free from family and work, where they can be protagonists of their lives, without putting themselves behind others:

*Sport serves not only for social integration but also for the general well‐being of people, even more so for immigrants. It is the last things that immigrants look for when they arrive in a new place, whatever kind of immigration we are talking about, from the unluckiest to the luckiest one. Sport is always the last thing one goes looking for (…) so seeing all the girls engaged in this activity was very nice because there was not only work, taking children to school, going to the supermarket, worrying about other commitments but also taking care of themselves and their commitment in the tournament*. (Female leader)

Regarding physical well‐being, the trust dimension provides equal opportunities for immigrants to play soccer within a safe space where discriminatory behaviors are sanctioned and where they can make a contribution to the team. As a result, they can concentrate on the play and the competition, and this energizes them, as noted by a female leader:

*I feel that my body is not 40 years old, I can play, I can train, so when I play soccer, I don't feel tired (…) playing makes me feel better because I feel that my body is ready, healthy… if in your life you are under pressure at work, in the family or whatever, when you play you feel at ease and, after you have played, you become energetic and positive. When you get home, you say, “okay, my body is ready, I play soccer and I am useful*. (Female leader)

Regarding interpersonal well‐being, the trade dimension allows immigrants to meet people who believe in and help them. Indeed, receiving confidence from staff members of the NGO makes them feel valued and esteemed. BM is configured as a space where reciprocal experiences of recognition are built, as reported by one participant:

*Balon makes me feel better because someone gives you confidence (…) a human being loves to be recognized as a worker, doing anything, on a personal level, in a deep way, not on an economic level* (Food volunteer)

Finally, regarding community well‐being, the art dimension helps immigrants develop cultural intelligence—that is, the ability to understand other people's ways of reading and interpreting situations based on the culture in which they are embedded. This ability is useful when establishing relationships with Italians and people of other countries. Participants feel that BM is a space where openness between people from different countries is encouraged and the knowledge of different customs and traditions, including Italian ones, is spread: “the tournament for us is a beautiful meeting, because you can meet people you've never seen, never met and never known, Latina or non‐Latina” (Female leader); “Since I have been here, I met many Italian people, they have often taught me the Italian language” (Female leader).

Developing cultural intelligence has also to do with understanding the connotation given to certain words in Italy, which may differ from that of other countries:

In Italy they think “*negro*” is a negative word. I have my daughter saved in the phone book as “*negra*”, my brother‐in‐law I call “*negro*”, my father used to call my mother “*negra*”. But it's a way to say love, sweetheart, it's a very sweet word. But in the pitch people think that one is denigrating someone, and we had to adapt because we are in Italy. (Male leader)

Finally, participants reported that they met other compatriots, recovering with them some traditions that they had lost during their lives in Italy:

*I have everything here, my husband and I both have a good job, we feel very well integrated. Now we also have a group of Brazilian friends so we prepare beans, barbecues, and other things that connect us to our country (…) Balon Mundial allowed me to recover my roots, which is something that I realize now how important it is. Because as much as we fit in—work, family, friends, everything you need in life, right? We had that. But to have a group of people that you know and that you can talk to in the same language and also recover nostalgic memories of your country, that's wellness too, it makes us feel good. I really enjoyed it*. (Female leader)

## 4 DISCUSSION

The present research focused on immigrant sport participation conceptualized as a specific type of community participation that uses sport as a tool to be part of the community. We aimed at exploring the creation of a psychological SOC within a soccer event and identifying how the psychological SOC is connected to subjective well‐being among immigrants who participate.

Specifically, sport participation in BM developed a psychological SOC by providing immigrants with a safe space—based on norms of accessibility and fair play—where they can mutually share traditions and form trusting bonds. The opportunity for immigrants to have access to spaces where no documentation, money or professional competence are needed is highly relevant. Many community psychologists (Gele & Harsløf, 2012; Martinez‐Damia et al., 2021; Salami et al., 2019) as well as sport researchers (Baker‐Lewton et al., 2016; Spaaij, 2013; Spaaij et al., 2019) have pointed out barriers that immigrants face in their participation. These impediments are especially related to access to structured club‐based sport (transport, lack of resources, policy gaps and competitiveness between organizations) and interpersonal (social disconnection, under‐valued rights, and mistrust), sociocultural (lack of ethno‐cultural diversity and discrimination) and personal constraints (lack of time, health and knowledge regarding the sport system). Providing accessible spaces to immigrants is the first step in promoting their participation. Moreover, the NGO board's strong attention to fair play made it possible to create and maintain a positive climate during the matches and impacted the formation of positive relationships between the teams. This particularity of BM makes the tournament different from other similar soccer events and sustains the formation of a common ground where people feel safe to share significant aspects and traditions of their country of origin. Immigrants who participate in the tournament said they had not perceived discrimination in their life in Italy. This could be because of the positive experiences within the tournament that have a “halo effect” on the experiences outside it. This effect may be a protective mechanism through which immigrants protect their subjective well‐being. At the same time, this effect could discourage immigrants from taking action to achieve fairer conditions. Hamidi (2003), for example, found that immigrants' desire to have fun together may decrease their motivation to fight for social justice.

We found that having a clear objective (i.e., winning the tournament) within an inclusive and supportive community (i.e., the team) is connected to a strong psychological SOC (McMillan, 1996), which is also connected to the enhancement of subjective well‐being across different domains (physical, psychological, interpersonal, and community). As supported by Bailey (2005), sport itself has the intrinsic advantage of providing groups with a common goal to achieve, and this generally sustains the formation of internal cohesion. BM participants found in the tournament a reason for staying together and committing to something relevant to their own country. Winning the tournament is reported as the key objective of sport participation and described by participants as something that make them proud and happy. As a consequence, we argue that sport participation can sustain immigrants' SOC because, specifically through sport participation and competition, immigrants are able to work and collaborate to accomplish a common task with their teams.

Sport participation in BM was based on national belonging. Participants report being proud to represent their own country within the tournament. Cultural homogeneity within the teams thus seems crucial for immigrants to sustain their SOC and well‐being. Furthermore, BM has the particularity of providing food banquets at the end of the matches. This is another characteristic that has been reported to sustain SOC, as food can be a powerful means to rediscover one's own origins, to share them with others and to discover new cultures and traditions. This allows for the development of a wider SOC not only within the team but across the tournament. Through food, it is possible to promote a dialogue among participants from diverse countries and to build shared stories around the tournament among players of different teams.

Finally, the results support those of studies that have connected community participation, psychological SOC and subjective well‐being (Alfieri et al., 2019; Garcia‐Cid et al., 2020; Liu, 2019; Ramos et al., 2020; Sonn, 2002). We found aspects of psychological SOC to be related to subjective well‐being. Indeed, immigrants engaging in this football event reported: (a) belongingness and ethnic pride, which is connected to happiness and life satisfaction; (b) social support, which is connected to creating important relationships and developing intercultural competence; and (c) symbols and positive narratives around immigrants in Italy, which are connected to feeling better and more accepted within the new country.

This study has some limitations. First, the usage of deductive thematic analysis. An inductive approach would have allowed for additional information not previously planned in our code book to be identified. Second, we did not include Italians in our sample. Even if this was a reasonable choice—as our aim was to explore immigrants' experience—we believe that future studies should also consider the perception of the host community regarding the sport experience to explore the similar or dissimilar dynamics that may affect it. It would be interesting to have some insights regarding the experience of nationals who participate in this type of event and to describe the cultural exchange that can happen.

## 5 CONCLUSION AND PRACTICAL IMPLICATIONS

The case of BM shows that sport participation needs specific structural characteristics to support SOC and well‐being among immigrants, including: open access to sport, the presence of specific sport norms sustaining a respectful climate among players, the presence of spaces and time beyond the matches for people to spend time together and create histories and rituals to share, and cultural homogeneity as a criterion for forming the teams. These findings have several practical implications. First, it is crucial to provide easy access to sport activities among immigrants, as they can be an effective means for the development of SOC and well‐being. Second, it is important to establish rules to create positive relationships. Nevertheless, although the dimensions of amusement and happiness were crucial in terms in terms of personal well‐being, they may create a “halo” effect on the perception of discrimination among immigrants outside the sport context. For this reason, there is the need to create spaces for reflection and development of critical thinking to recognize the injustices experienced outside of the playing field. Finally, the research underlines the importance of supporting the building of ethnically homogeneous teams, as they create cultural pride and a stage where immigrants can show themselves in a positive light. This finding is partially in contrast with the conclusions of some authors (Hatzigeorgiadis et al., 2013; Müller et al., 2008), who reported that sport events enhance the sense of intra‐group belonging but discourage the openness of immigrant communities with respect to the host community. We did not find the risk of teams isolating themselves from the Italian culture due to the creation of culturally homogeneous groups within the BM tournament. Nevertheless, we argue that it is important to create spaces of interconnection where the different cultures can get to know one another and start to understand and appreciate the culture of the new country.

This study focused on a small group of immigrants in Northern Italy, but it strongly touches on a pressing global need for local communities to support immigrants, who mainly experience structural and interpersonal marginalization. Indeed, Italy maybe seen as an example of how European societies have generally related to the immigration phenomenon. Racial prejudice, discrimination, difficulties for immigrants to access employment (ENAR, 2017), and negative narratives in the media (Eberl et al., 2018) have been detected in many other European countries as well as Italy. Therefore, it is crucial to find ways to facilitate intergroup exchange, and sports participation may be a very promising tool, as it can be largely accessible, engaging and experienced universally across diverse ethnic communities.
